# Psychotropic drugs and the environment: a comprehensive analysis of surface water concentrations and associated risks

**DOI:** 10.1192/j.eurpsy.2023.1582

**Published:** 2023-07-19

**Authors:** J. Luykx

**Affiliations:** MUMC+, MUMC+, Maastricht

## Abstract

**Introduction:**

For most countries it is currently unknown to what degree concentrations of psychotropic drugs in surface water exceed environmental threshold concentrations (ETCs) [MOU1] for ecosystems and what risk mitigation could be applied. ETCs are defined as per-compound threshold concentrations above which detrimental effects on reproduction, growth, and mortality of aquatic organisms cannot be exclude

**Objectives:**

To quantify levels of antidepressants, antipsychotics, mood stabilizers, and benzodiazepines in surface water, investigate their sources and assess whether these levels exceed ETCs.

**Methods: Design:**

Cross-sectional analysis of measured and modeled data. Environmental levels were compared to ETCs to evaluate their risks for the aquatic environment. Finally, sources of psychotropic drugs were investigated.

**Setting:**

All available Dutch water monitoring data from all regional and national monitoring campaigns of 2019, the last year before the COVID-19 pandemic.

**Exposures:**

Concentrations of aripiprazole, carbamazepine and its metabolites, clozapine, diazepam, (es)citalopram, fluoxetine, haloperidol, nortriptyline, olanzapine, oxazepam, temazepam, quetiapine, sertraline, valproic acid, and venlafaxine.

**Main outcomes and measures:**

The main outcomes were measured and modeled concentrations of the aforementioned agents in surface water. As a secondary outcome, where possible, average risk quotients (RQs) were calculated by dividing the measured or modeled concentrations by the ETC. An RQ > 1 was interpreted as a risk to the environment.

**Results:**

Psychotropic drug samples (n=1201; 14-520 measurements per drug) showed the highest average concentrations for oxazepam (0.91 μg/L; RQ = 1.89) and carbamazepine (0.74 μg/L; RQ = 1.48), with individual measurements exceeding ETCs. For other drugs, measured concentrations did not reach the detection limit (amisulpride, (es)citalopram, quetiapine, and venlafaxine) or did not exceed the ETC (fluoxetine). Furthermore, households contributed most to psychotropics in surface water. Finally, psychotropics were cleared less efficiently from a wastewater treatment plant than other medications.

**Conclusions:**

Psychotropic drugs are present in surface water, are primarily emitted by households, and may put organisms at risk. We signal a need to reduce concentrations of several psychotropic agents in the environment. Our findings set the stage for policies and research aimed at curtailing emissions of psychotropic drugs into the environment and highlight a need for responsible prescribing and waste measures.

**Disclosure of Interest:**

None Declared

